# Partial Nephrectomy as Management of Oligometastatic Cancer with Limited Systemic Treatment Options: A Case Report

**DOI:** 10.15586/jkc.v12i3.414

**Published:** 2025-09-10

**Authors:** Chibuzor Victor Nwachukwu, Christopher Michael Brede, Gerald Paul Wright, Brian Robert Lane

**Affiliations:** 1 Department of Surgery, Michigan State University College of Human Medicine, Lansing, MI, USA;; 2 Division of Urology, Corewell Health Medical Group West, Grand Rapids, MI, USA;; 3 Department of Surgical Oncology, Corewell Health Medical Group West, Grand Rapids, MI, USA

**Keywords:** Renal metastasis, melanoma, melanoma metastasis, systemic therapy, Immunotherapy, kidney cancer

## Abstract

Ocular melanoma is a form of melanoma that rarely offers actionable mutations for treatment with systemic therapy and is relatively radioresistant. As such, surgery is the mainstay of treatment for localized disease and can be considered for oligometastatic disease. We present a case of ocular melanoma that recurred with a solitary renal metastasis 9 years after initial diagnosis and treatment with intraocular brachytherapy. After multidisciplinary discussion, the patient underwent a partial nephrectomy for her solitary renal metastasis. The patient continued in follow-up 3.5 years after partial nephrectomy. She was treated again surgically for a solitary metastasis to the breast before initiation of systemic therapy once multifocal disease was identified. We suggest interdisciplinary management of patients with metastatic involvement of target organs, given the rapidly changing treatment landscape for melanoma and other forms of cancer.

## Introduction

Melanoma has been identified as the fifth most common cancer in males and females (1). While most common in the skin, melanoma can also arise in the eyes, as in this patient, or on mucosal surfaces (1). When occurring in the skin, melanomas spread superficially for many years before growing vertically past the basement membrane and invading the dermis due to stepwise accumulation of genetic abnormalities (2). Although melanoma frequently metastasizes, most commonly to the skin and subcutaneous tissue, metastases to the kidney are extremely rare (3). There have been a few prior cases reported in the literature, with radical nephrectomy (RN) performed for renal metastasis in every case (4–9). With changing practices for renal malignancy, specifically the increased use of partial nephrectomy (PN) when technically feasible, it is unclear that these cases reflect what currently should be offered in the management of metastatic melanoma to the kidney. National Comprehensive Cancer Network (NCCN) guidelines for melanoma suggest surgery for localized cancer and systemic therapy for metastatic disease. Prognosis for patients with metastatic melanoma has improved greatly with the use of immune checkpoint inhibitors, such as ipilimumab and nivolumab, as well as other molecular-targeted therapies. Before the dawn of immune-targeted therapies, data from a randomized control trial showed a benefit of combined surgery and systemic therapies compared to systemic therapies alone (10). Due to the gains made in systemic therapies for metastatic melanoma, the role of surgery for metastatic disease has become less clear-cut and is often assessed on a case-by-case basis (11–14).

Ocular melanoma consists of melanomas in the uveal tract (iris, ciliary body, choroid) and conjunctiva. Local treatment of uveal melanomas is known to prevent recurrence in 95% of cases, but still has a 50% risk of metastasizing to other sites due to micrometastasis and variable latency period (15). Most metastases occur within 5–7 years (16). Secondary uveal cancers are usually metastasized from a primary lung or breast cancer, while primary uveal melanoma may metastasize to the liver, which is why liver MRI is obtained on uveal melanoma diagnosis and follow-up (17). Since uveal melanomas are relatively radio-resistant, high-dose radiation or plaque brachytherapy are commonly used as treatment options (18).

Management of metastases to the kidney has been studied for multiple types of nonrenal primary malignancy, such as lung and colorectal, and cancer of unknown origin. Data are from largely small and medium-sized case series, rather than clinical specialists managing cancer types with more renal malignancies who may have more clinical experience with partial versus radical nephrectomy decisions, but in the melanoma space, this is the first discussion of PN.

Based on the limited prior reported experience with management of melanoma metastasis to the kidney (ocular and nonocular), we present the management of a patient with metastatic uveal melanoma to the kidney that was managed differently than in any prior report and conclude contemporary management.

## Case Presentation

**Figure 1:**
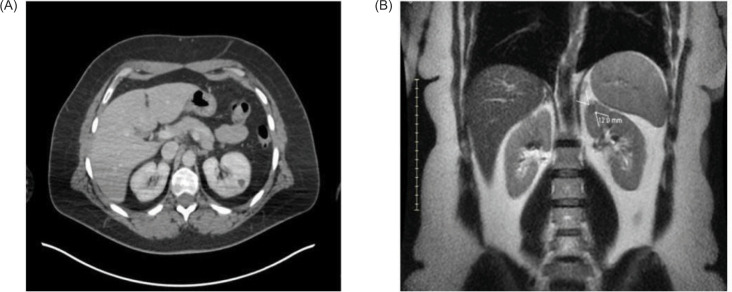
Small renal mass in the upper pole of the left kidney as seen on CT and MRI. (A) Axial view from CT demonstrates a 12 mm lesion. (B) Coronal view from the abdominal MRI also shows a solitary 12 mm upper pole anterior endophytic lesion of the left kidney. Pathology indicates metastatic ocular melanoma.

The patient was initially diagnosed with a tumor of the choroid of the right eye, identified pathologically by immunohistochemistry as malignant melanoma. MRI of the abdomen at the time of diagnosis showed the liver, kidneys, and adrenals were within normal limits. The patient was treated with plaque brachytherapy, 21 seeds and an 18 mm plaque left in for 4 days. The patient remained in remission for 9 years until the detection of a 1.5 cm soft tissue nodule in the anterior upper pole of the left kidney on routine surveillance imaging. The patient presented with no symptoms, no hematuria, flank pain, or weight loss. Whole body PET-CT scan showed no other suspicious lesions, and physical exam revealed no cutaneous lesions. Recurrence of melanoma to the left kidney was confirmed, and renal cell carcinoma (RCC) was ruled out by percutaneous renal mass biopsy (Figure 1). After consultation with urology and surgical departments, and medical oncology, the decision was made to pursue surgical removal of this solitary melanoma metastasis. The tumor was of low complexity, with a RENAL score of 6 (R1 E3 N1 L1), for which AUA guidelines indicate PN to be appropriate (19). The patient underwent robotic partial nephrectomy with final pathology confirming melanoma with immunochemistry positive for SOX-10 (20). PN had the same level of difficulty as a nonmelanoma renal mass of similar size. The operative time was 2.5 h, and the length of hospital stay was 3 days. To date, the patient has been monitored with serial imaging every 6 months. No adjuvant systemic therapy was given post-nephrectomy, as the patient had no other evidence of disease at that time, and systemic options for ocular melanoma are limited.

**Figure 2:**
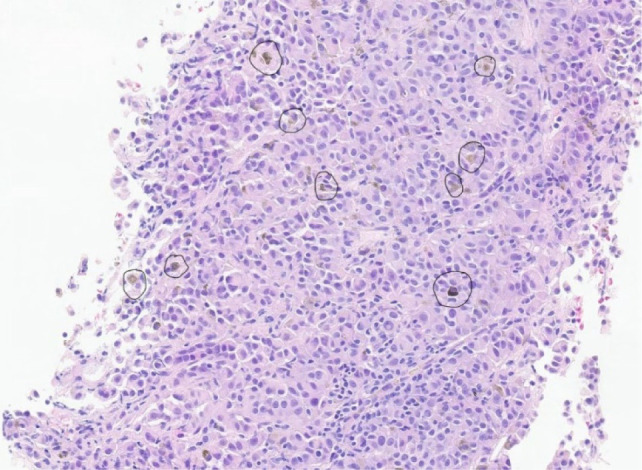
This is a histopathological image of the metastatic melanoma showing pigmented, atypical cells with abundant melanin (in dark circles). The tumor cells appear brown to black against the background of eosinophilic (pink-purple) H and E stain.

She was without recurrence or evidence of further metastatic disease for 1 year, when she was noted to have a solitary breast metastasis on a routine mammogram. She elected to have excision of the breast lesion without radiation or systemic therapy after multidisciplinary discussion with the breast cancer team. Pathology demonstrated metastatic melanoma with negative margins. The patient was again disease-free for about 1 year, when she developed new pulmonary and hepatic lesions on surveillance imaging. She was again brought before an interdisciplinary tumor board, which recommended initiation of systemic therapy. Subsequent cancer progression occurred, and she eventually passed away from her malignancy 12 years after initial diagnosis and 3.5 years after kidney surgery (Figure 2).

## Discussion

Metastatic melanoma to the kidney can pose a significant diagnostic challenge for the pathologist as it can histologically mimic both low- and high-grade primary renal neoplasms (21). This may explain the similarities of 20th-century treatment options for these historically poor responsive malignancies (RCC and melanoma). There are no clear guidelines on the management of the patient with rare metastatic involvement of the kidney by melanoma, but previous literature has shown one consistent treatment modality, RN. For secondary involvement of the kidney by metastases from other types of malignancies, the therapeutic approach depends on whether the metastasis is solitary or multiple, the nature of the primary tumor, and whether there is widespread metastasis to other organs (21). Secondary involvement of the kidney by nonrenal tumors can occur as an isolated solitary lesion, and in some patients, the kidney can be the first and only site of metastatic involvement (22). When an RN is selected without prior renal mass biopsy, patients may experience unnecessary kidney loss if severe symptoms, such as intractable hematuria and/or pain, are not present.

Retrospective analysis of cases in which isolated synchronous or metachronous hematogenous metastases were resected, surgical resection as part of an aggressive multidisciplinary approach positively correlated to favorable outcomes, along with complete control of the primary site, confirmed solitary metastatic disease, good performance status, and metachronous lesions (23). In another large retrospective series with 161 patients of multiple cancer types, the median overall survival was higher in patients treated with surgery (median years from primary tumor diagnosis: 4.8 versus 3.1 years and from time of renal metastatic diagnosis 2.2 versus 1.1 years). They concluded that surgical intervention in carefully selected patients with oligometastatic disease and good performance status should be considered (24).

For metastatic melanoma with distant metastases, recommendations depend on whether the metastases are limited or extensive. Although melanoma used to have very limited options, a wide array of new options makes management more complicated, and the role of interdisciplinary decision-making and treatment should now be similar to contemporary management of other aggressive nonhematologic malignancies. When limited metastases are present, appropriate considerations include observation, resection, irradiation, or ablation of metastases, systemic therapy, or a combination of target-directed and systemic therapies. With more widespread disease, hospice or palliative management versus systemic therapy with or without treatment of specific targets is reasonable. First-line systemic therapy includes: (1) anti–programmed cell death protein 1 (PD-1) monotherapy with pembrolizumab or nivolumab; (2) nivolumab/ipilimumab; and (3) a combination of dabrafenib plus trametinib, vemurafenib plus cobimetinib, or encorafenib plus binimetinib if the tumor contains a BRAF V600–activating mutation (19). Second-line therapies include anti-PD-1 monotherapy, targeted therapy if a BRAF V600 is present, ipilimumab, high-dose interleukin-2 (IL-2), cytotoxic agents, or imatinib for tumors with activating mutations of KIT (19).

For patients with oligometastatic disease, treatment of the gross site(s) of disease has the potential benefits of delaying or preventing the need for systemic therapy, reducing the disease burden (cytoreduction), and/or addressing symptoms caused by the metastases. Management of metastatic melanoma to the kidney depends on the extent of the disease overall and the best estimates of life expectancy. There have been no randomized trials that compare treatment of oligometastases versus systemic therapy in this disease space. A multivariate analysis showed that surgical resection of metastasis may prolong survival in certain patients with distant metastasis from intraocular melanoma (25). So, in oligometastatic disease in a patient with limited systemic options, metastasectomy is certainly an option worth considering. Ocular melanoma (choroidal, uveal, etc.) is a distinctly different entity from cutaneous melanoma. It is typically not responsive to systemic therapies such as immune checkpoint inhibitors and rarely offers actionable mutations, such as BRAF V600E, with high-dose radiation as the mainstay treatment due to being relatively radioresistant (18). So, the rationale for surgery here was primarily oligometastatic disease with limited systemic treatment options.

For renal surgery for oligometastatic nonrenal cancer, the same principles should apply for resection of RCC, with nephron-sparing surgery if feasible. The AUA guidelines address the appropriateness of PN and RN, based on tumor size, complexity, and other patient characteristics (26, 27). PN was preferred here as this was not a high-complexity tumor necessitating challenging PN even in experienced hands. For such tumors in patients without preexisting CKD, a normal contralateral kidney, and predicted eGFR after RN of greater than 45 mL/min/1.73m^2^, RN is preferred. But for tumors < 4 cm undergoing surgery, PN is selected in > 90% of the cases. The value of PN is the preservation of functioning renal parenchyma, which results in better renal function compared to RN (27). For patients with widespread disease, resection of a renal metastasis can provide palliative benefit when symptoms are significant, but otherwise is not generally recommended.

Extrarenal metastasis to the kidney is very rare, with incidences ranging between 2.4 and 12.6% and was historically described in autopsy series (28), without standard treatment regimens. However, they are seen more in contemporary literature due to better CT and MRI imaging (21), with the majority of renal metastases coming from the lungs, breast, and GI tracts. The previous literature indicates that renal melanoma is initially treated surgically (4–9). Subsequent systemic treatment with chemotherapy or radiation is reserved for cases with metastasis beyond the kidneys (7). All prior reports of renal surgery for metastatic melanoma reported RN even in cases of solitary small renal masses that may have been amenable to PN. This could be a result of the comfort level of the surgeons involved, as PN use has risen steadily over the last two decades, and the absence of guidelines for the management of melanoma metastasis to the kidney. We hope that management in this case will lead others to pursue multidisciplinary discussion with the most appropriate surgery (or other treatment) applied for cases such as this. Patients with metastatic disease have even more to benefit from renal function-preserving treatments, since some treatments are renotoxic and/or renally excreted, having a prespecified GFR > 60 or 45 mL/min/173m^2^ is necessary to receive others, clinical trials often mandate adequate renal function based on GFR, and/or subsequent comorbidities for which having preserved renal function will assist in recovery. As literature regarding the benefits of cytoreductive PN expands (29), we hope this discussion assists clinical teams in considering the benefits of PN for nonrenal malignancies as well.
